# Phase II Trial of Romidepsin as Consolidation Therapy after Gemcitabine, Dexamethasone, and Cisplatin in Elderly Transplant-Ineligible Patients with Relapsed/Refractory Peripheral T-Cell Lymphoma

**DOI:** 10.3390/hematolrep16020034

**Published:** 2024-05-28

**Authors:** Satoshi Yamasaki, Hiroatsu Iida, Akio Saito, Morio Matsumoto, Yoshiaki Kuroda, Tohru Izumi, Akiko M. Saito, Hiroaki Miyoshi, Koichi Ohshima, Hirokazu Nagai, Hiromi Iwasaki

**Affiliations:** 1 Department of Internal Medicine, Kyushu University Beppu Hospital, 4546 Tsurumihara, Tsurumi, Beppu 874-0838, Japan; 2 Department of Hematology, Clinical Research Institute, NHO Kyushu Medical Center, Fukuoka 810-0065, Japan; iwasaki.hiromi.vr@mail.hosp.go.jp; 3 Department of Hematology and Oncology Research, NHO Nagoya Medical Center, Nagoya 460-0001, Japan; iida.hiroatsu.kr@mail.hosp.go.jp (H.I.); akiko.saito@nnh.go.jp (A.M.S.); nagai.hirokazu.uf@mail.hosp.go.jp (H.N.); 4 Department of Hematology, NHO Shibukawa Medical Center, Shibukawa 377-0204, Japan; saito.akio.hf@mail.hosp.go.jp (A.S.); matsumoto.morio.zr@mail.hosp.go.jp (M.M.); 5 Department of Hematology, NHO Hiroshimanishi Medical Center, Otake 739-0696, Japan; kuroda.yoshiaki.hx@mail.hosp.go.jp; 6 Department of Hematology, NHO Sendai Medical Center, Sendai 983-8520, Japan; izumi.toru.fz@mail.hosp.go.jp; 7 Department of Pathology, School of Medicine, Kurume University, Kurume 830-0011, Japan; miyoshi_hiroaki@med.kurume-u.ac.jp (H.M.); ohshima_kouichi@med.kurume-u.ac.jp (K.O.)

**Keywords:** peripheral T-cell lymphoma not otherwise specified, angioimmunoblastic T-cell lymphoma, transplant-ineligible, older, gemcitabine, cisplatin, romidepsin

## Abstract

Romidepsin is an important therapeutic option for patients with peripheral T-cell lymphoma (PTCL). However, the timing of romidepsin administration remains controversial. The objective of this study was to characterize the safety and efficacy of romidepsin as consolidation therapy after gemcitabine, dexamethasone, and cisplatin (GDP) therapy (GDPR). This study of patients treated between March 2019 and March 2021 was registered with the Japan Registry of Clinical Trials (registration number: jRCT0000000519). If complete response, partial response, or stable disease was confirmed after 2–4 GDP cycles, romidepsin was administered every 4 weeks for 1 year. Seven patients with relapsed/refractory (R/R) PTCL (T-follicular helper phenotype [n = 1] and angioimmunoblastic T-cell lymphoma [n = 6]) were included in this prospective study (PTCL-GDPR). After a median follow-up of 34 months of patients in PTCL-GDPR, the 2-year overall survival rate was 71%, and the overall response rate after treatment was 57%. Common adverse events in patients with PTCL-GDPR included hematological toxicities such as neutropenia, which improved with supportive treatment. There were no treatment-related mortalities. GDPR might be safe and effective in elderly transplant-ineligible patients with R/R PTCL; however, further investigation is required.

## 1. Introduction

With the increasing number of older adults worldwide, the average age of patients with hematopoietic malignancies is expected to increase every year. One disease for which old age is a particularly poor prognostic factor is lymphoma [1]. Additionally, the risk of treatment-related mortality is increased in elderly patients. Old age increases the likelihood of various disease factors and patient factors, including a history of other diseases and organ disorders, resulting in a poor response to chemotherapy.

Recently, brentuximab vedotin plus cyclophosphamide, doxorubicin, and prednisone therapy was found to provide a superior overall survival (OS) rate versus cyclophosphamide, doxorubicin, vincristine, and prednisone (CHOP) therapy as an initial treatment in both CD30+ peripheral T-cell lymphoma not otherwise specified (PTCL-NOS) and angioimmunoblastic T-cell lymphoma (AITL) [2,3]. However, there are no standard treatments for elderly transplant-ineligible patients with relapsed/refractory (R/R) PTCL.

Previous reports described the effectiveness of gemcitabine, dexamethasone, and cisplatin (GDP) therapy in patients with R/R PTCL, but this regimen has been used in only a small number of cases [4,5]. The 60–70% response rate for GDP therapy is promising, but its median disease-free survival remains unsatisfactory at 9–10 months. To date, no treatment methods with the potential to prolong OS have been developed, and studies focusing on the prolongation of OS as the primary endpoint are difficult to conduct.

In Japan, only single-agent romidepsin, a histone deacetylase inhibitor approved for R/R PTCL, administration is covered by insurance and considered safe. Phase I/II trials on romidepsin, conducted in Japan, found that romidepsin caused few critical side effects other than temporary anemia, and despite its single-agent nature, the overall response rate (ORR) was 25% (15% complete response [CR] rate) [6]; romidepsin demonstrated similar efficacy and tolerability for R/R PTCL regardless of age [7]. Although romidepsin maintenance after allogeneic hematopoietic stem cell transplantation (HSCT) for AITL was promising [8], no clinical trials have examined consolidation therapies following salvage chemotherapy in elderly transplant-ineligible patients with R/R PTCL. Although a standard treatment has not been established in R/R PTCL and the endpoint in most clinical trials was ORR [9] rather than OS, future developments in treatment may be facilitated by introducing romidepsin.

We performed this study for R/R PTCL in elderly transplant-ineligible patients as a non-randomized confirmatory trial to characterize the safety and efficacy of romidepsin as consolidation therapy (GDPR) following conventional salvage chemotherapy (GDP), from which only short-term effects can be expected.

## 2. Materials and Methods

### 2.1. Prospective and Retrospective Study Design

This was a phase II multicenter open-label, single-arm trial conducted at 18 Japanese centers belonging to the Japanese National Hospital Organization between March 2019 and March 2021. The inclusion criterion was the receipt of CHOP as induction chemotherapy. The exclusion criteria were as follows: (1) any history of solid cancers or hematological malignancies before diagnosis; (2) receipt of autologous SCT; and (3) donor unavailability for allogeneic HSCT. The enrolled patients were followed until death or the end of follow-up. The study was conducted in accordance with institutional guidelines and the principles of the Declaration of Helsinki. The protocol was approved by the institutional review boards of the 18 participating institutions. The protocol details of the prospective study [10] were published previously. We examined the safety and efficacy of GDPR as salvage chemotherapy for elderly transplant-ineligible patients with R/R PTCL-NOS or AITL according to the 2016 World Health Organization classification [11], and a pathology review was performed centrally by an expert hematopathologist. The dose-adjustment paradigm was designed to reduce age-related or non-hematological toxicities. The trial was approved by the ethics boards of all participating centers, and written informed consent was provided by all participants. An independent data and safety monitoring committee monitored the trial every 6 months. The Japanese National Hospital Organization had its own financial support. This study was registered in the Japan Registry of Clinical Trials (registration number: jRCT0000000519).

### 2.2. Treatment Protocol of the Prospective Study

Eligible patients aged 65 years or older with R/R PTCL who had received at least one standard chemotherapeutic regimen (CHOP) were included. Baseline assessments included physical examination; standard laboratory tests; computed tomography (CT) of the chest, abdomen, and pelvis; and bone marrow biopsy if indicated. Eligible patients were required to have measurable disease by CT or physical examination, an Eastern Cooperative Oncology Group performance status of 0–2, and acceptable hematological and biochemical parameters. Patients were excluded if they had previously received treatment with gemcitabine and cisplatin or if they had central nervous system involvement with PTCL; a history of hepatitis B virus, hepatitis C virus, human T-cell virus type 1 virus, or human immunodeficiency virus infection; or a medical condition that would interfere with the safe administration of the protocol chemotherapy.

The protocol commenced within 4 weeks after patient registration. The attending physician decided whether each patient should undergo inpatient or outpatient treatment. GDP therapy was administered every 3 weeks. Gemcitabine was administered intravenously at a dose of 1000 mg/m^2^/day on days 1 and 8, dexamethasone was administered orally or intravenously at a dose of 40 mg/day on days 1–4, and cisplatin was administered intravenously at a dose of 75 mg/m^2^/day on day 1. After two courses of GDP therapy, an interim response evaluation was conducted via positron emission tomography (PET)–computed tomography or CT. The evaluation criteria in the Revised Response Criteria for Malignant Lymphoma [12] were used to evaluate tumor shrinkage. In cases of complete response (CR), partial response (PR), or stable disease (SD), two additional courses of GDP therapy were administered. However, if the doctor in charge determined that the patient did not have the required tolerance for reasons unrelated to disease status, no additional GDP therapy was added, or only one additional course was provided. Patients who were unable to achieve a result equal to or better than SD before the interim response evaluation after the two courses of GDP therapy (progressive disease [PD]) and those who were confirmed as having PD during the treatment protocol stopped treatment at that point. Patients who completed the planned GDP therapy underwent a final response evaluation via CT and PET-CT after the conclusion of the final course.

Patients deemed to have achieved CR, PR, or SD based on the response evaluation after the completion of GDP therapy received romidepsin every 4 weeks.

One course comprised romidepsin 14 mg/m^2^ administered intravenously once per day on days 1, 8, and 15. A final response evaluation was performed 6 months after the end of treatment, which was continued for 12 courses over the course of one year.

Treatment was designed to be delivered to patients in an outpatient setting, and a recommended minimum hydration schedule was included for cisplatin. Each participating center was responsible for determining policies for supportive care after treatment with dose-adjusted GDP. Granulocyte colony-stimulating factor (G-CSF) was given daily if neutrophil counts decreased to less than 1000/μL.

### 2.3. Efficacy Evaluation

Post-therapy follow-up and observation included a medical examination and blood test once every 3 months, and diagnostic imaging (CT) and an evaluation of the intercurrent lesions present at the time of registration were assessed once every 6 months. During follow-up, the presence or absence as well as the severity of adverse events (AEs) was evaluated and recorded on each patient’s medical chart.

The efficacy evaluation conducted after the end of treatment consisted of a medical examination and blood test once every 3 months and diagnostic imaging (CT) and evaluation of the intercurrent lesions present at the time of registration once every 6 months. The absence or presence of a relapse, deterioration, or secondary cancer was clinically assessed during all other times.

Disease status assessments included physical examinations, CT, and bone marrow analysis. Tumor response evaluation was performed according to the Revised Response Criteria for Malignant Lymphoma [12]. CT of the neck, thorax, abdomen, and pelvis or PET-CT was performed every 3 months for up to 24 months or until the initiation of alternative PTCL treatment, whichever came first. The primary endpoint was the 2-year OS rate, which was measured from the time of diagnosis until the date of death from any cause as described previously [13]. Patients who had not relapsed, progressed, or died were censored at the date of the last follow-up. OS was calculated using the Kaplan–Meier method and compared using the log-rank test. AEs from the start of treatment until 6 months after the last treatment were graded according to the Common Terminology Criteria for Adverse Events (CTCAEs) version 4.0. Quality of life (QOL) was measured using the QOL Questionnaire for Cancer Patients Treated with Anticancer Drugs (QOL-ACD) [14] and the SF-36^®^ health survey [15]. The patients were assessed at baseline, during the middle of treatment, at the end of the protocol, and 6 months after the end of the protocol. A statistically significant change in the QOL score versus baseline was considered a clinically meaningful change.

### 2.4. Efficacy Evaluation Following the Conclusion of Treatment

The efficacy evaluation following the end of treatment consisted of a medical examination and blood test once every 3 months and diagnostic imaging (CT) and evaluation of the intercurrent lesions present at the time of registration once every 6 months. The absence or presence of relapse, deterioration, or secondary cancer was clinically assessed during all other times. In patients with grade ≥ 3 AEs according to CTCAEs version 4.0 during treatment, the dose of each chemotherapeutic drug in the subsequent cycle was reduced, and the protocol regimen (0%, 25%, 50%, 75%, or 100% dose) was delayed at the physician’s discretion.

SF-36^®^, a comprehensive survey assessing patient health, and QOL-ACD, a QOL measurement tool specifically for patients with cancer, were used to survey QOL. To determine the necessary expenses, the total estimated charges on all the patients’ bills associated with the delivery of treatment from the start of therapy until the final response evaluation were summed.

### 2.5. Statistical Analysis

The efficacy analysis set consisted of eligible patients receiving protocol treatment after registration. The safety analysis set was defined as all patients receiving protocol treatment after registration. The incidence of AEs was calculated. OS was calculated using the Kaplan–Meier method. Changes from baseline in the QOL-ACD and SF-36^®^ scores were determined by the Wilcoxon signed-rank test. The significance level was 0.05 in both one- and two-tailed tests. Analyses were conducted using EZR (Saitama Medical Center, Saitama, Japan; http://www.jichi.ac.jp/saitama-sct/SaitamaHP.files/statmedEN.html, accessed on 10 December 2023) [16], which is a graphical user interface for R (The R Foundation for Statistical Computing, version 4.2.2; www.r-project.org, accessed on 10 December 2023) and a modified version of R commander (version 2.8–0) designed to add statistical functions. The full analysis set (FAS) included all registered patients. Regarding the protocol rules for treatment and combination therapy, the set, excluding patients from the FAS who violated the eligibility or exclusion criteria or prohibited concomitant medication or prohibited combination therapy, was labeled as the per-protocol set. However, patients who seriously violated the protocol (e.g., not obtaining approval or seriously violating trial procedures) were excluded. Among the registered patients, the group excluding those who did not undergo any treatment was termed the safety analysis set.

Regarding efficacy evaluations, FAS was the main analysis set. For response rates, the point estimate and an 80% confidence interval were calculated. OS was estimated using the Kaplan–Meier method. All safety analyses were performed relative to the safety evaluation set. For AEs and serious AEs, the point estimate of the proportion of occurrence was calculated. Regarding SF-36 and QOL-ACD, any temporal changes were summarized.

## 3. Results

### 3.1. Patient and Clinical Characteristics

**Figure 1:**
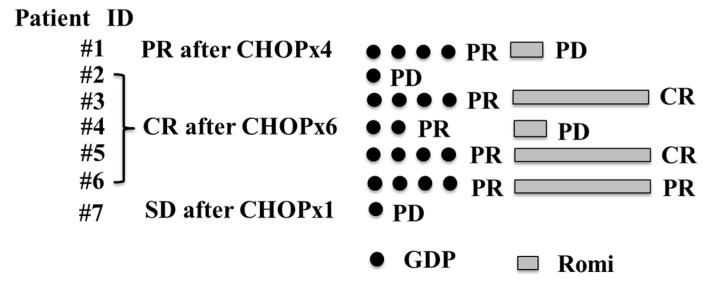
Flow diagram of patients in the prospective trial (PTCL-GDPR) who received GDP plus Romi for PTCL. PTCL, peripheral T-cell lymphoma; GDP, gemcitabine, dexamethasone, and cisplatin; Romi, romidepsin; CR, complete response; PR, partial response; SD, stable disease; PD, progressive disease; CHOP, cyclophosphamide, doxorubicin, vincristine, and prednisone.

Seven patients older than 65 years with R/R transplant-ineligible PTCL were enrolled from 18 hospitals in this prospective study (PTCL-GDPR, Figure 1). This study was terminated in March 2022 to comply with the planned enrollment period. All diagnoses of biopsies were reclassified and confirmed as PTCL by an experienced hematopathologist. No patients received high-dose chemotherapy followed by autologous SCT because of the physician’s decision based on their advanced age.

The baseline characteristics of the participants are presented in Table 1. In the prospective study, the median patient age was 74 years (range, 72–82 years), all patients were male, most patients had stage III (n = 4) or IV (n = 3) disease, most patients had a high-intermediate International Prognostic Index (IPI, n = 5), two patients failed to achieve CR with initial CHOP therapy, and five patients experienced relapse of lymphoma after 1 year of initial CHOP therapy. Six patients diagnosed with AITL and one patient diagnosed with PTCL T-follicular helper phenotype were assessed.

### 3.2. Treatment Outcomes

**Figure 2:**
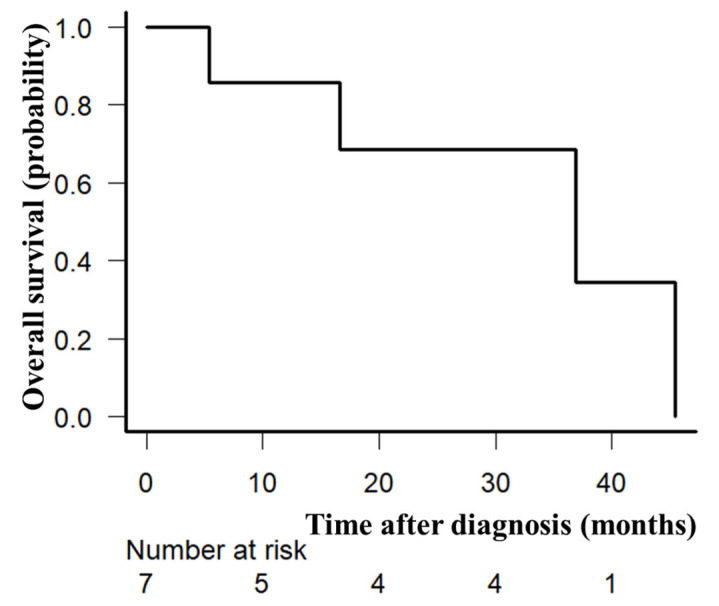
Overall survival. Overall survival of seven evaluable patients enrolled in the prospective trial (PTCL-GDPR) was plotted using the Kaplan–Meier method. The 2-year overall survival rate was 71%.

At least one cycle of protocol therapy was completed in seven patients. The median number of GDP cycles was four (range, 1–4). The ORR after PTCL-GDPR treatment was 57% (n = 4). Two of the patients had an improved response to romidepsin. The CR rate after PTCL-GDPR was 29% (n = 2). Regarding the disease status at study entry, the ORR after initial CHOP was 86% among patients with relapse more than 1 year after initial CHOP (the CR rate after initial CHOP was 71%), and the primary induction failure rate after initial CHOP was 14%. There were no patients with relapse less than 1 year after the initial CHOP in this study. After a median follow-up of 34 months (range, 5–46) after PTCL-GDPR, the 2-year OS rate was 71% (Figure 2). Lymphoma progression was the main cause of death, and four (57%) patients died of lymphoma.

### 3.3. Toxicity Assessments, QOL, and Cost

Seven patients constituted the safety analysis set. All AEs including grade 3 and 4 events are summarized in Table 2. Common AEs included hematological toxicities such as neutropenia, which improved with supportive treatment. There were no treatment-related mortalities. Although every patient received G-CSF, gemcitabine was omitted in two patients (29%) on day 8 in cycle 1. Cardiac arrest after febrile neutropenia occurred in one patient after initial GDP therapy. Two patients discontinued GDPR after one cycle of GDP because of lymphoma and died of lymphoma. In patients with grade ≥ 3 AEs, romidepsin treatment was stopped, the dose of romidepsin in the subsequent cycle was reduced, and the protocol regimen was delayed at the physician’s discretion.

**Figure 3:**
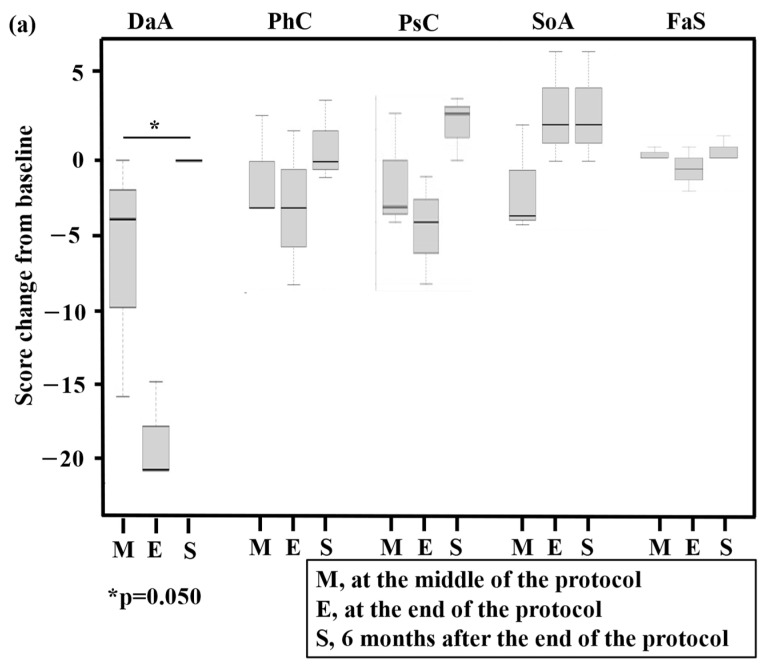

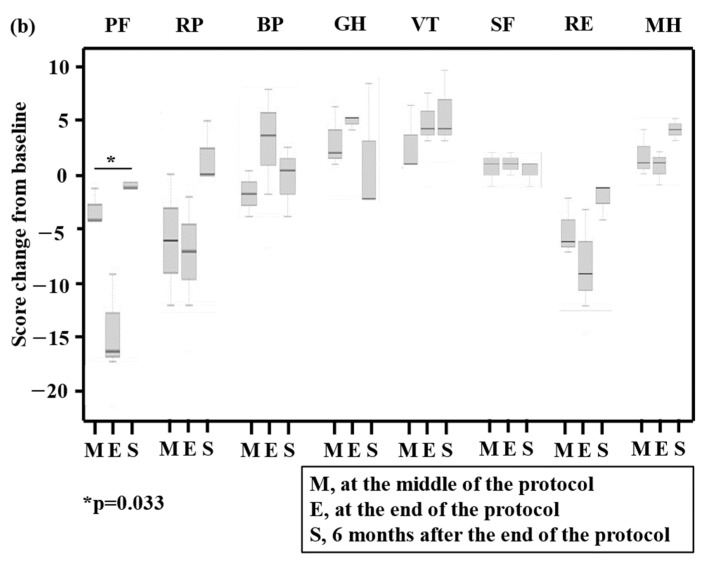
Box and whisker plot of total quality of life (QOL) scores using QOL-ACD (**a**) and SF-36^®^ (**b**) for three patients who completed the prospective trial (PTCL-GDPR). The bottom and top of the box are the 25th and 75th percentiles, respectively. The thick band and the square near the middle of the box are the 50th percentile (the median) and mean, respectively. The ends of the whiskers represent the lowest datum still within 1.5-fold of the interquartile range (IQR) of the lower quartile and the highest datum still within 1.5-fold of the IQR of the upper quartile. QOL was assessed at baseline, in the middle of the protocol (M), at the end of the protocol (E), and 6 months after the end of the protocol (S). A statistically significant change (*p*-value) in a QOL score compared with the baseline score is presented in the figures. QOL-ACD, QOL Questionnaire for Cancer Patients Treated with Anticancer Drugs; N, number of patients; DaA, daily activity; PhC, physical condition; PsC, psychological condition; SoA, social attitude; FaS, face scale; PF, physical functioning; RP, role limitations caused by physical health problems; BP, bodily pain; GH, general health perception; VT, vitality; SF, social functioning; RE, role of limitations caused by personal or emotional health problems; MH, general mental health.

Regarding QOL assessments using QOL-ACD and SF-36^®^, changes from baseline are presented in Figure 3a,b (three patients completed GDPR therapy) and the Supplementary Materials, Figure S1a,b (total, n = 6), respectively. Regarding QOL-ACD in patients who completed GDPR, significant worsening at the end of the protocol and improvement at 6 months after the end of the protocol were found for the daily activity score (*p* = 0.050). Regarding SF-36^®^ in patients who completed GDPR, significant worsening at the end of the protocol and improvement at 6 months after the end of the protocol were found for the physical functioning score (*p* = 0.033). The mean cumulative total direct medical costs were JPY 1.2 million (range, JPY 1.1–1.2 million).

## 4. Discussion

To the best of our knowledge, this is the first study to evaluate GDPR after initial CHOP for elderly transplant-ineligible patients with R/R PTCL. This study supported the efficacy and safety of GDPR in elderly transplant-ineligible patients with R/R PTCL. Results comparable to or better than those of conventional regimens using GDP [4,5], romidepsin [6,17], or gemcitabine plus romidepsin [18] were anticipated in patients receiving GDPR in this study.

Median OS after GDP therapy in patients with PTCL has been reported to range from 9 to 10 months. Romidepsin has the potential to be effective against tumors other than hematopoietic malignancies. Hence, we expect romidepsin to influence treatment development and exhibit efficacy against other cancerous tumors such as acute lymphocytic leukemia [19]. Two studies stated that the median duration of response for romidepsin was 11.1 months [6,17]; thus, we hypothesize that 12 months of treatment is acceptable in elderly patients. It is important to comprehensively conduct medical evaluations by considering clinical factors, QOL, and financial aspects. To date, we have been able to obtain information only on the effects of therapy selection on patients’ QOL and financial circumstances, the latter of which is difficult to verify. Hematological toxicities, which are common AEs of GDPR, did not affect the QOL outcome. Although the number of patients and events was extremely small, the results of this clinical study are expected to provide information supporting the establishment of better therapies for patients with PTCL in the future [20].

Older age is reportedly associated with a poor prognosis among patients with PTCL, including PTCL-NOS and AITL [21]. This is believed to be associated with the increased risk of treatment-related mortality in older patients. Older age affects disease and patient factors, and older patients’ responsiveness to chemotherapeutic treatments is low [1]. In a previous study, the addition of romidepsin to CHOP for patients with previously untreated PTCL did not improve progression-free survival, response rates, or OS, and was associated with an increased frequency of grade ≥ 3 treatment-emergent AEs [22]. However, the conditions in elderly patients with PTCL are often complicated by previous illnesses and organ damage. Increasing the treatment intensity, as is carried out for younger patients, may therefore be more difficult in older patients with PTCL [1]. As such, sequential use of single-agent romidepsin, such as GDPR, may be more beneficial for older patients with R/R PTCL.

We reported that IPI, which is the most used score to predict survival in patients with PTCL-NOS [10] and the best score for predicting CR after six or more courses of CHOP or CHOP-like therapy among transplant-ineligible patients with PTCL-NOS or AITL, was useful for investigating the risk factors associated with outcomes. In this study, we could not evaluate the risk factors including IPI that influence OS, which explains why these data could not be used to evaluate the risk factors for identifying suitable patients for GDPR because of the small number of patients.

This study had multiple limitations. We evaluated OS in patients with R/R PTCL with the two major subtypes, namely PTCL-NOS and AITL. A decrease in treatment intensity might have been the cause of poorer treatment outcomes in elderly patients versus younger patients. The effects of different salvage therapies were unclear because of the limited range of available salvage therapies for PTCL-NOS and AITL, and the salvage therapies after R/R disease were determined by physician and patient preferences. In addition, because allogeneic HSCT prolongs survival according to the recommendations in the guidelines [23], the reasons for autologous SCT ineligibility were not categorized. Some patients did not undergo autologous SCT because of patient or physician choice, so they were not strictly “transplant-ineligible” because of age or comorbidities, which might have resulted in bias, potentially exemplified by older age and poor outcome risks, as previously noted [24]. There was thus the potential for selection bias in relation to ineligibility for autologous SCT because no proper randomization could be achieved. Finally, the sample size and short follow-up duration might have limited our ability to analyze the outcomes of GDPR.

In conclusion, PTCL-NOS and AITL are rare conditions associated with poor prognoses in patients who develop relapse or disease progression after initial chemotherapy. However, GDPR might be safe and effective for elderly transplant-ineligible patients with R/R PTCL, and further investigation is warranted. In addition, new treatment strategies for PTCL-NOS and AITL could allow the identification of individuals suitable for novel therapies, such as combined oral 5-azacytidine and romidepsin [25].

## Figures and Tables

**Table 1:** Characteristics of patients.

Characteristic		n = 7
Median age (range), years		74 (72–82)
≥75 years of age, n (%)		3 (43)
Sex, n (%)	male	7 (100)
	female	0
ECOG PS, n (%)	0	2 (29)
	1	5 (71)
	2	0
Ann Arbor stage, n (%)	I	0
	II	0
	III	4 (57)
	IV	3 (43)
BM involvement, n (%)		2 (29)
IPI, n (%)	low	0
	low–intermediate	1 (14)
	high–intermediate	5 (71)
	high	1 (14)
Median No. (range) of cycles of initial CHOP		6 (1–6)
Radiotherapy after initial CHOP, n (%)		0
Best response after initial CHOP, n (%)	CR	5 (71)
	PR	1 (14)
	SD	1 (14)

n, total number of patients assessed; ECOG PS, Eastern Cooperative Oncology Group performance status; BM, bone marrow; IPI, International Prognostic Index; CHOP, cyclophosphamide, doxorubicin, vincristine, and prednisone; CR, complete remission; PR, partial remission; SD, stable disease.

**Table 2:** Grade ≥3 adverse events in patients in the prospective study.

	PTCL-GDPR										
	GDP				Romidepsin						
Toxicities/Cycles	1	2	3	4	1	2	3	5	7	10	12
	n = 7	n = 5	n = 4	n = 4	n = 5	n = 5	n = 5	n = 3	n = 3	n = 3	n = 3
Hematological toxicities, n (%)											
WBC decreased	5 (71)	3 (60)	2 (50)	2 (50)	3 (60)	2 (40)	2 (40)	1 (33)	1 (33)	0	0
Neutrophil count decreased	5 (71)	3 (60)	2 (50)	2 (50)	3 (60)	2 (40)	2 (40)	1 (33)	1 (33)	0	0
Anemia	2 (29)	2 (40)	0	0	1 (20)	0	1 (20)	0	0	0	0
Thrombocytopenia	0	3 (60)	1 (25)	1 (25)	0	0	0	0	0	0	0
VZV infections, n (%)	0	0	0	0	0	0	0	0	0	1 (33)	0
Non-hematological toxicities, n (%)											
Gastrointestinal											
Anorexia	0	0	0	0	0	0	0	0	0	0	1 (33)

PTCL-GDPR, prospective study; GDP, gemcitabine, dexamethasone, and cisplatin; WBC, white blood cell; VZV, varicella-zoster virus.

## Data Availability

The Institutional Review Board of our 18 institutions in the National Hospital Organization group in Japan does not allow open access. However, upon reasonable request, additional analyses can be performed after contacting the corresponding author.

## Supplementary Materials

The following supporting information can be downloaded at: https://www.mdpi.com/article/10.3390/hematolrep16020034/s1, Figure S1: Box and whisker plot of total quality of life (QOL) scores using QOL-ACD (a) and SF-36 (b) in six evaluable patients in the prospective trial (PTCL-GDPR).
