# Supplementary material for: Phase II Trial of Romidepsin as Consolidation Therapy after Gemcitabine, Dexamethasone, and Cisplatin in Elderly Transplant-Ineligible Patients with Relapsed/Refractory Peripheral T-Cell Lymphoma
Source: Hematol Rep. 2024 May 28;16(2):336–46. doi: 10.3390/hematolrep16020034 (PMC11204088; doi:10.3390/hematolrep16020034)
Supplement: Supplementary file 1 [file hematolrep-16-00034-s001.zip › hematolrep-2791842-supplementary.pdf]

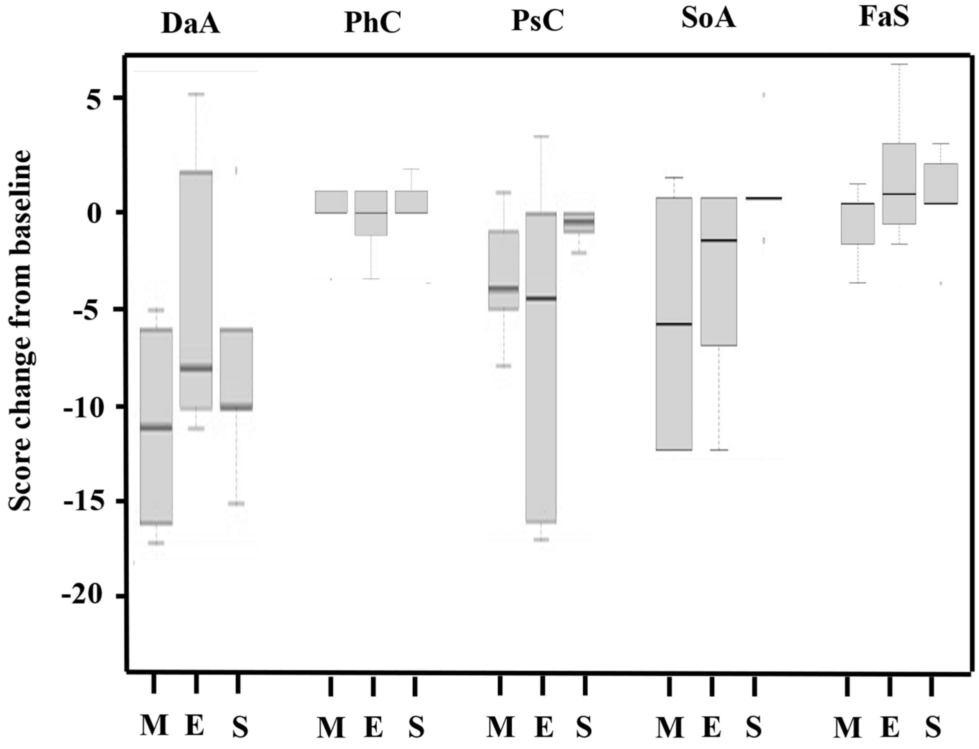

a

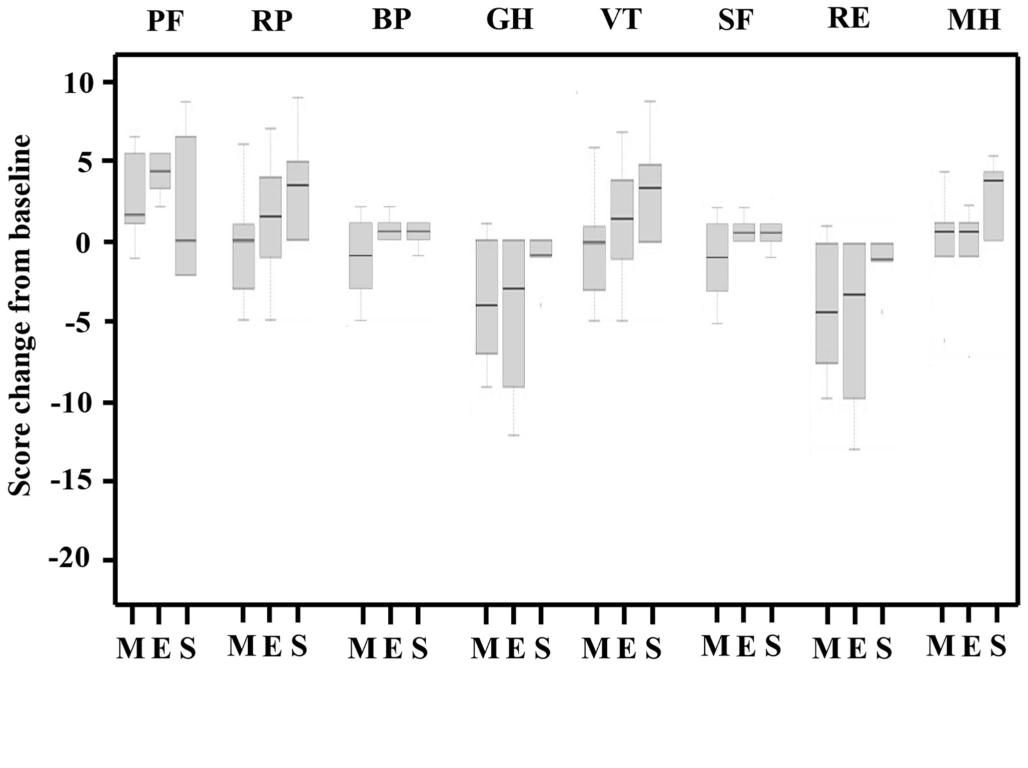

b

Supplemental Figure S1. Box and whisker plot of total quality of life (QOL) scores using QOL-ACD (a) and SF-36 (b) in six evaluable patients in the prospective trial (PTCL-GDPR). The bottom and top of the box are the 25th and 75th percentiles, respectively. The thick band is the 50th percentile (median). The ends of the whiskers represent the lowest datum still within 1.5-fold of the interquartile range (IQR) of the lower quartile and the highest datum still within 1.5-fold of the IQR of the upper

quartile. The open circles are outliers between 1.5- and 3-fold of the IQR from the end of a box. QOL was assessed at baseline, during the middle of chemotherapy (M), at the end of chemotherapy (E), and 6 months after the end of chemotherapy (S). *A statistically significant change in a QOL score compared among the scores for M, E, and S. QOL-ACD, QOL Questionnaire for Cancer Patients Treated with Anticancer Drugs; BP, bodily pain; DaA, daily activity; FaS, face scale; GH, general health perception; MH, general mental health; PF, physical functioning; PhC, physical condition; PsC, psychological condition; RE, role of limitations caused by personal or emotional health problems; RP, role limitations caused by health problems; SF, social functioning; SoA, social attitude; VT, vitality.
